# Intraoperative optical coherence tomography (I-OCT) assisted passage of the encircling element during retinal detachment surgery

**DOI:** 10.3205/oc000197

**Published:** 2022-04-01

**Authors:** Ashish Markan, Nikita Gupta, Basavaraj Tigari, Ramandeep Singh, Mohit Dogra

**Affiliations:** 1 Advanced Eye Centre, Postgraduate Institute of Medical Education and Research, Chandigarh, India

**Keywords:** microscope integrated OCT, retinal detachment surgery, encircling element, vitreoretina fellows, scleral sutures

## Abstract

**Objective:** To describe the role of microscope integrated optical tomography (Mi-OCT) in passage of the encircling element during retinal detachment surgery.

**Materials and methods:** Mi-OCT was switched on while passing scleral anchoring sutures to secure the encircling element. The depth of the suture tract was seen in real time while the needle was passed through the sclera. The amount of scleral intend achieved was assessed using Mi-OCT at the end of the surgery.

**Results:** The depth of needle tract through the sclera and the amount of scleral indentation achieved while tying the sutures could be successfully analyzed using Mi-OCT.

**Conclusion:** Mi-OCT can be used as an adjunct while training vitreoretinal fellows and trainees in surgical procedures like passage of scleral sutures for anchoring the encircling element.

## Background

Microscope integrated optical coherence tomography (Mi-OCT) provides real-time cross sectional images of tissues during various ophthalmic surgeries, thereby helping surgeons in achieving optimal results [1], [2], [3]. Utility of Mi-OCT goes beyond mere diagnostics as it influences intraoperative decision making by altering critical surgical steps in 43% of anterior segment surgeries and about 30% of posterior segment surgeries [4]. Mi-OCT has been used in ophthalmic surgeries like lamellar corneal procedures, cataract and refractive surgeries, glaucoma surgeries and vitreoretinal surgeries [5], [6], [7], [8], [9]. This report highlights the role of intraoperative OCT in aiding the passage of 5-0 dacron (polyethylene terephthalate) partial-thickness scleral sutures used to anchor the encircling band during retinal detachment surgery and assessing the amount of scleral indent achieved. Utilization of advanced surgical adjuncts like Mi-OCT, especially in academic institutes with multiple vitreo-retinal fellows and trainees, during surgical training can help both the mentor as well as the trainee assess adequate suture depth. This would potentially avoid complications like inadvertent scleral perforation and high or low buckle indentation.

## Case description

**Figure 1:**
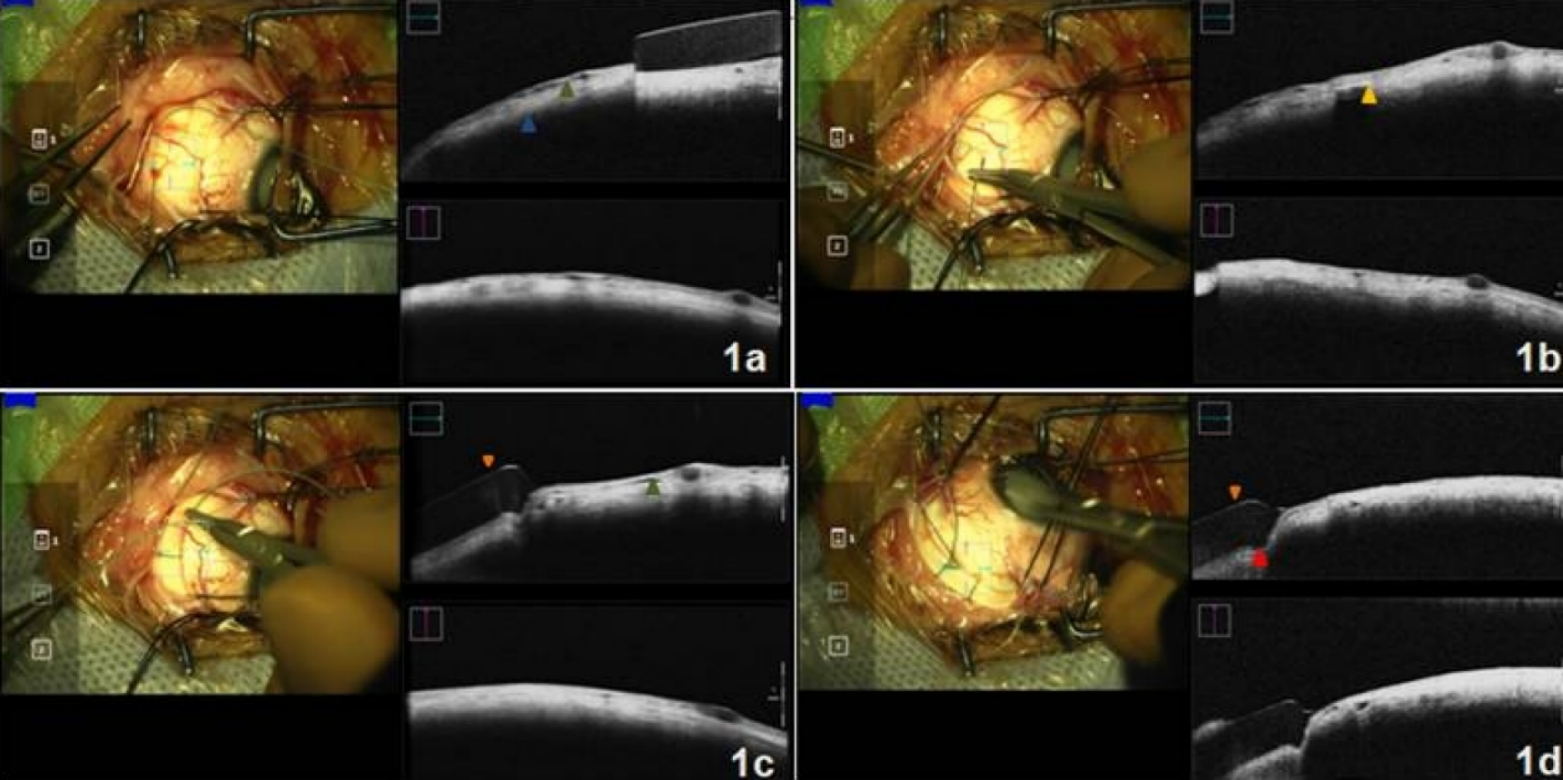
a) Realtime passage of suture through the sclera, seen as hyporeflective space (green arrowhead). Sclero-choroidal junction can be easily appreciated on Mi-OCT (blue arrowhead). b) Hyperreflective band with backshadowing (yellow arrowhead) corresponding to real time passage of suture needle. c) Encircling 240 band (orange arrowhead) and hyporeflective space corresponding to depth of suture tract (green arrowhead). d) Desired scleral intend (red arrowhead) achieved by tying the scleral sutures.

A 10-year-old male presented with diminution of vision in his right eye (OD) for the past 5 months. Examination revealed a white cataract with no view of the fundus. Total retinal detachment was detected on ocular ultrasonography. Pars plana lensectomy (PPL) with pars plana vitrectomy (PPV) along with an encircling 240 scleral band was planned. After performing a 3600 conjunctival peritomy and bridling of all the recti with 3-0 silk suture, a 240 encircling element (Labtician Ophthalmics, Oakville, Canada) was passed underneath all the recti muscles. This was followed by passage of 5-0 dacron (polyethylene terephthalate) sutures (Aurolab, Madurai), 12mm from the limbus in all quadrants, to secure the encircling 240 band. Mi-OCT (The RESCAN 700, Carl Zeiss Meditec, Germany) was switched on while passing the needle through the sclera to assess the depth of the suture bite. Mi-OCT was also used to assess the amount of scleral indentation achieved after the mattress sutures were tied. Figure 1a (Fig. 1) shows the presence of a hyporeflective space (green arrowhead), which corresponds to the passage of suture through the sclera. This helps the surgeon assess and titrate the desired depth of the suture through the sclera. Full-thickness of sclera and sclera-choroidal interface (blue arrowhead) was well appreciated using intraoperative OCT. Figure 1b (Fig. 1) shows a hyperreflective band (yellow arrow head) with back shadowing which corresponds to the real-time passage of the needle through the sclera. Figure 1c (Fig. 1) shows a hyporeflective band (orange arrowhead) which corresponds to the encircling 240 scleral band. Passage of suture can be appreciated as hyporeflective space in the sclera (green arrowhead). Similarly mattress sutures were passed in all four quadrants. The desired scleral indentation (red arrowhead) was achieved by tying the scleral sutures (Figure 1d (Fig. 1)).

## Discussion

Inadvertent scleral perforation during passage of sutures for scleral explants is one of the most dreaded complications. It may lead to choroidal hemorrhage, subretinal hemorrhage, vitreous hemorrhage, retinal and/or vitreous incarceration and iatrogenic retinal break formation [10]. Mi-OCT can help guide vitreo-retinal trainees and novice surgeons while passing partial-thickness mattress scleral sutures for anchoring encircling elements and performing scleral buckling surgeries, thereby minimizing complications. Another benefit is titration of the amount of scleral indent achieved with the scleral explant by ensuring the desired length and depth of scleral sutures based on real-time feedback provided by Mi-OCT.

## Conclusion

Mi-OCT plays an important role in intraoperative decision making in various important surgical procedures and can help surgeons, especially trainees and fellows during their initial surgical learning.

## Abbreviations

- Mi-OCT: Microscope integrated optical coherence tomography
- PPV: Pars plana vitrectomy

## Notes

### Informed consent

Informed consent has been obtained from the patient for the publication of this case report.

### Competing interests

The authors declare that they have no competing interests.
